# Complementary role of computed tomography texture analysis for differentiation of pancreatic ductal adenocarcinoma from pancreatic neuroendocrine tumors in the portal-venous enhancement phase

**DOI:** 10.1007/s00261-020-02406-9

**Published:** 2020-01-17

**Authors:** Christian Philipp Reinert, Karolin Baumgartner, Tobias Hepp, Michael Bitzer, Marius Horger

**Affiliations:** 1 Department of Diagnostic and Interventional Radiology, University Hospital Tübingen, Hoppe-Seyler-Str. 3, 72076 Tübingen, Germany; 2 Department of Internal Medicine I, Hepatology, Gastroenterology, Infectiology, University Hospital Tübingen, Otfried-Müller-Str. 10, 72076 Tübingen, Germany

**Keywords:** Tomography, X-ray computed, Neuroendocrine tumors, Carcinoma, pancreatic ductal, Pancreatic neoplasms

## Abstract

**Purpose:**

To assess the role of CT-texture analysis (CTTA) for differentiation of pancreatic ductal adenocarcinoma (PDAC) from pancreatic neuroendocrine neoplasm (PNEN) in the portal-venous phase as compared with visual assessment and tumor-to-pancreas attenuation ratios.

**Methods:**

53 patients (66.1 ± 8.6y) with PDAC and 42 patients (65.5 ± 12.2y) with PNEN who underwent contrast-enhanced CT for primary staging were evaluated. Volumes of interests (VOIs) were set in the tumor tissue at the portal-venous phase excluding adjacent structures. Based on pyradiomics library, 92 textural features were extracted including 1st, 2nd, and higher order features, and then compared between PNEN and PDAC. The visual assessment classified tumors into hypo-, iso-, or hyperdense to pancreas parenchyma or into homogeneous/heterogeneous. Additionally, attenuation ratios between the tumors and the non-involved pancreas were calculated.

**Results:**

8/92 (8.6%) highly significant (*p* < 0.005) discriminatory textural features between PDAC and PNEN were identified including the 1st order features “median,” “total energy,” “energy,” “10th percentile,” “90th percentile,” “minimum,” “maximum,” and the 2nd order feature “Gray-Level co-occurrence Matrix (GLCM) Informational Measure of Correlation (Imc2).” In PNEN, the higher order feature “GLSZM Small Area High Gray-Level Emphasis” proved significantly higher in G1 compared to G2/3 tumors (*p* < 0.05). The tumor/parenchyma ratios as well as the visual assessment into hypo-/iso-/hyperdense or homogeneous/heterogeneous did not significantly differ between PDAC and PNEN.

**Conclusions:**

Our data indicate that CTTA is a feasible tool for differentiation of PNEN from PDAC and also of G1 from G2/3 PNEN in the portal-venous phase. Visual assessment and tumor-to-parenchyma ratios were not useful for discrimination.

**Electronic supplementary material:**

The online version of this article (10.1007/s00261-020-02406-9) contains supplementary material, which is available to authorized users.

## Introduction

CT-texture analysis (CTTA) is an emerging field of investigation capable to identify specific tissue features meant to more accurately characterize tumors and other tissue types (e.g., inflammatory disorders) by using image data quantification [1, 2]. Results of CTTA can be further tested for correlations with other non-image-based patient data (laboratory, genetic, etc.) in order to allow for a more individualized approach of these patients and their diseases, to provide valuable information capable of stratifying prognosis, and search for the most appropriate therapy [3–5].

Pancreatic ductal adenocarcinoma (PDAC) is the most frequent pancreatic malignancy with dismal prognosis if not amenable for primary surgery [6]. CT is the most frequently used imaging technique due to its excellent spatial resolution, tissue contrast, and availability. The primary aims of imaging are tumor detection and delineation, assessment of vascular invasion, and distant seeding. However, primarily non-invasive differentiation from other pancreatic neoplasms is also important. Among the less frequent solid pancreatic tumors (e.g., metastases, acinar cell carcinoma, papillary and solid tumor, adenoma, hamartoma, etc.) the neuroendocrine tumors are playing an increasingly important role due to their in part overlapping imaging findings to PDAC and different management options [7, 8]. Whereas PDAC are generally hypo- or isoattenuated compared to the adjacent pancreatic tissue in the portal-venous enhancement phase, PNEN usually are expected to be hyperattenuated [8]. Differentiation between these two pancreatic tumor entities requires generally a multi-phase examinational protocol as PNEN are best delineated on arterial phases due to their early and high amount of vascularization, whereas PDAC are inducing desmoplastic reaction experiencing mostly a late enhancement. However, many PDAC and PNEN patients undergo first portal-venous CT for elucidation of the cause of cholestasis (e.g., in case of a pancreatic head mass) and of other non-specific symptoms or the tumors are detected incidentally. In the portal-venous enhancement phase up to 42% of PDACs may be isoattenuated to the normal pancreatic tissue and in PNEN the contrast blush can already have fade away so that differentiation between the two may be challenging depending strongly on the applied examinational protocol (e.g., contrast agent volume, flow, delay time, etc.) [9]. Moreover, PNEN may also exhibit different attenuation values on portal-venous enhancement phase which correlates well with the intratumoral microvascular density, the amount of tumor stroma, and tumor grading [10, 11]. PNEN are expected to be homogeneous hypervascular followed by early wash-out in the venous phase due to their origin from the highly vascularized islet cells, which receive 10–20% of the entire pancreatic supply [12]. As progression towards malignancy is associated with derangement in vessel architecture and function, larger PNEN have a less homogenous hypervascular pattern and may show a delayed contrast enhancement [13].

Hence, considerable overlap in the mean attenuation of PDAC and PNEN exist in this enhancement phase. Moreover, the contrast between the tumor and the non-involved adjacent pancreatic tissue is dependent on the patient age and pancreas size and consistency [14]. Previous reports have addressed the issue of image data quantification in PNEN and PDAC using textural features [15–18]. They emphasized mainly the role of texture analysis for predicting tumor grading.

In this current retrospective evaluation of ninety-five patients with primarily diagnosis of PDAC and PNEN we aimed at defining CTTA-based imaging fingerprints for differentiation of these two tumor entities in the portal-venous enhancement phase using ninety-two representative features belonging to all statistical (1st, 2nd, and higher) orders.

## Materials and methods

### Patient characteristics

This was a retrospective analysis of 53 patients (66.1 ± 8.6y, 24 female) with PDAC and 42 patients (65.5 ± 12.2y; 18 female) with PNEN identified by a patient chart search at our institution between 09/2008 and 08/2018. Our institutional ethic board committee approved the retrospective data evaluation and registered this study under the number 140/2019BO2.

### CT-examination protocol

Computed tomography (CT) was performed with patients in the supine position using 128-slice MDCT scanners (SOMATOM Definition AS + or SOMATOM Definition Flash, Siemens Healthcare). All patients underwent contrast-enhanced CT in the portal-venous enhancement phase (60–70 s delay) using thin-slice image data acquisition. Following examinational parameters were used: 120 kV tube voltage, 200–250 mAs tube current, soft tissue image reconstruction kernel, and 1 mm slice thickness for image reconstruction. Weight-adapted iodine contrast agent was given intravenously at a rate of 2 mL/s followed by a 30 mL saline chaser. Image reconstruction was performed in all patients using filtered back projection.

### Computed tomography texture analysis (CTTA)

**Fig. 1:**
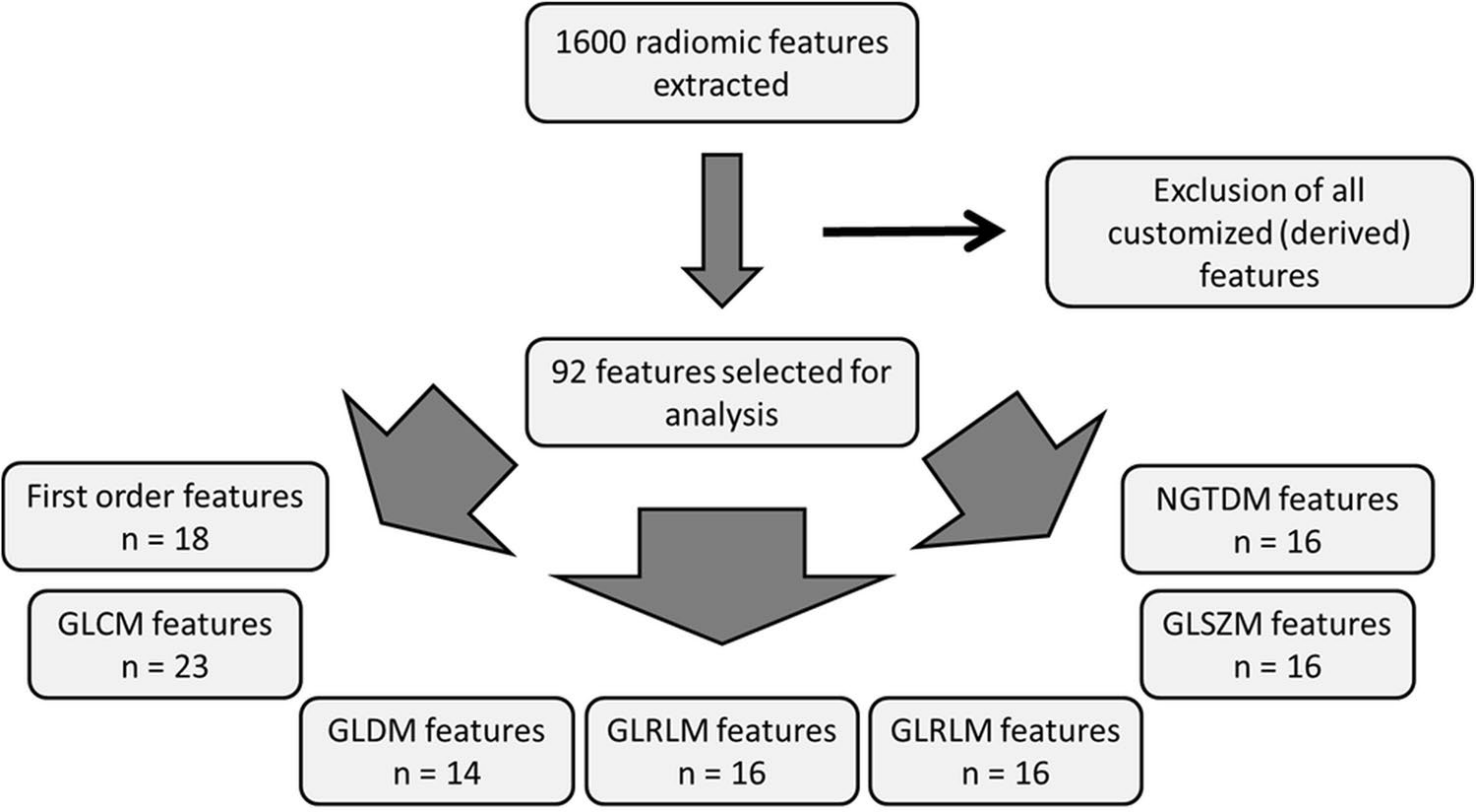
Textural feature selection

CTTA was performed using radiomics software (Siemens Healthcare) that is based on the pyradiomics package, a python package for the extraction of radiomics features from medical imaging [19]. A total of 1600 radiomic features were primarily extracted. However, in order to limit redundancy of some of the results derived, e.g., from the use also of customized (derived) features, we decided to restrict to the original 92 features including 18 first-order features, 23 Gray-Level Co-occurrence Matrix (GLCM) features, 14 Gray-Level Dependence Matrix (GLDM) features, 16 Gray-Level Run Length Matrix (GLRLM) features, 16 Gray-Level Size Zone Matrix (GLSZM) features, and 5 Neighboring Gray Tone Difference Matrix (NGTDM) features (Fig. 1).

**Fig. 2:**
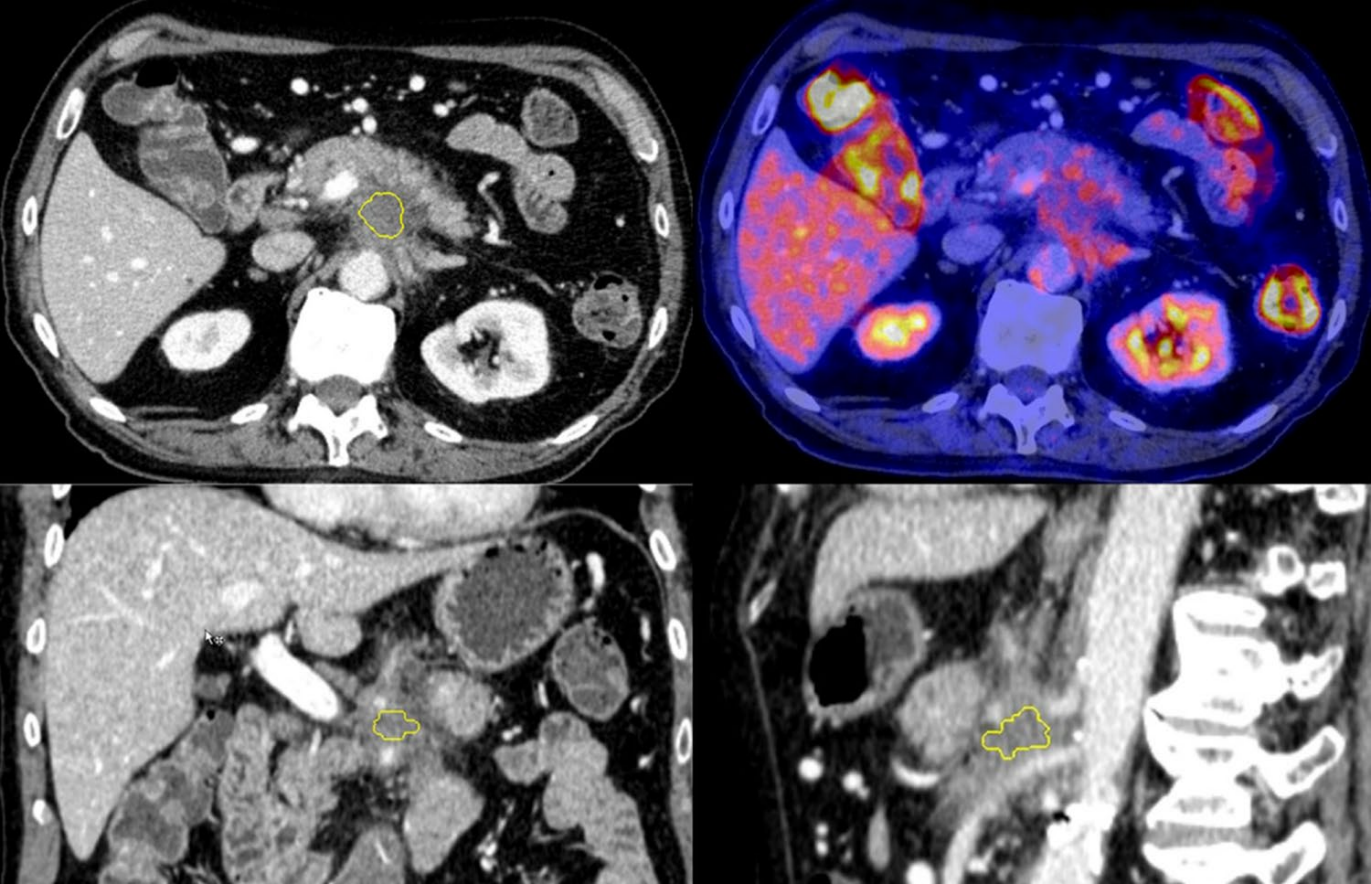
Tumor segmentation. A 80-year-old male patient with PDAC infiltrating the celiac trunk and showing only moderate tracer avidity in ^18^F-fluorodeoxyglucose (FDG) positron emission tomography/computed tomography (PET/CT) (right upper row). Three-dimensional tumor segmentation under exclusion of adjacent structures

CTTA was applied on image data sets that were reconstructed with 1 mm slice thickness. Volumes of interest (VOIs) were drawn freehand on the transversal portal-venous CT-image data using the largest cross-section diameter (Fig. 2). Neighboring tissue (e.g., blood vessels), calcifications as well as visible necrotic areas were carefully excluded. The procedure of VOI setting was performed by a senior radiologist with 25 years of experience in abdominal and oncologic imaging. To provide comparability for all data sets standardized measurements were performed. The computation of each texture type for an input volume of interest involved assigning a new value (“texture value”) to all voxels of that volume of interest and thus creating a “texture image.” In a first step, we performed image filtration for selectively extracting features of different sizes and intensity variations. In the second step, quantification of tissue radiomics was performed using a series of derived images displaying features at a fine spatial scale (2 mm in radius) within a volume of interest. Window ranges of 0–400 HU were used. Computation was performed on the current voxel and its neighborhood, and the results of that were stored as the texture value of the current voxel. This was repeated for every voxel in the volume of interest. The radiomics features used belonged to 1st order (energy, total energy, entropy, minimum, maximum, mean, median, interquartile range, mean absolute deviation, robust mean absolute deviation, standard deviation, skewness, kurtosis, variance, and uniformity); 2nd (gray-level co-occurrence matrix) order and higher order features (gray-level size zone matrix, gray-level run length matrix, neighboring gray-tone difference matrix, and gray-level dependence matrix), the latter including subfeatures described by the pyradiomics library (Supplementary material).

**Fig. 3:**
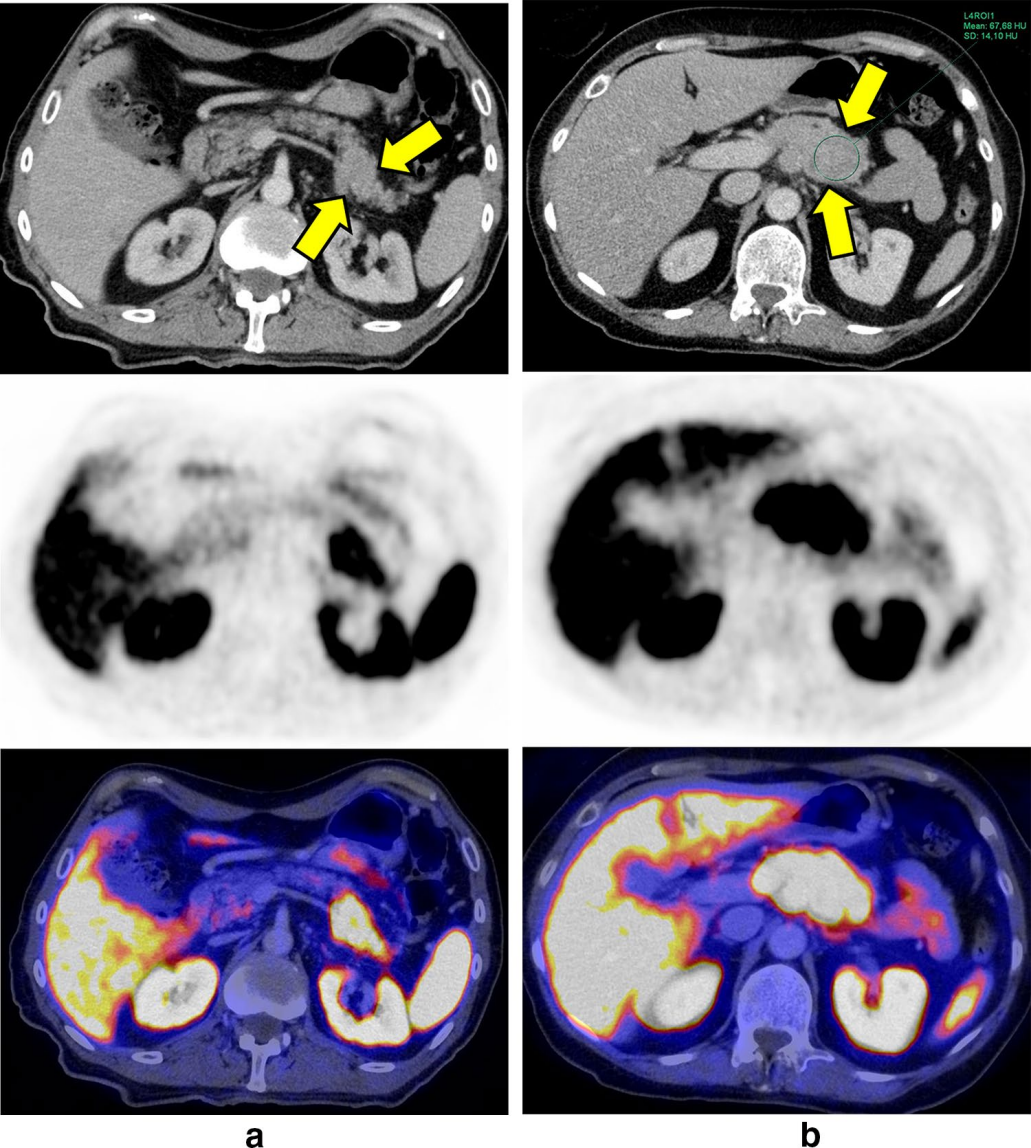
**a** 65-year-old patient with a G1 PNEN in the tail of the pancreas (yellow arrows). The tumor shows an increased radiotracer uptake in ^68^Ga-DOMITATE-PET. **b** 72-year-old patient with a G3 PNEN in the transition between pancreas body and tail. The tumor (yellow arrows) appears isodense (67.7 ± 14 HU) to the pancreas parenchyma (68.1 ± 8 HU). ^68^Ga-DOMITATE-PET reveals an increased radiotracer uptake of the tumor

Finally, we grouped the PNEN according to their grading (G2 + G3) versus G1 as well as PDAC G1 versus G2 versus G3 and applied again radiomics analysis trying to identify significant features enabling non-invasive evaluation of tumor grading (Fig. 3a).

### Visual tumor characterization and quantification of tumor-to-parenchyma ratios

16/53 patients with PDAC underwent ^18^F-FDG-PET/CT and 18/42 patients underwent ^68^Ga-DOMITATE-PET/CT for staging, in which the lesions could be identified due to increased radiotracer uptake and routinely performed arterial phase. The other patients received a dual phase CT (including an arterial phase). Additionally, 48/95 patients received a contrast-enhanced MRI of the pancreas. These information were used for placing large hand drawn ROIs both in the tumor tissue and in the non-involved pancreas parenchyma avoiding partial volume averaging. Attenuation values (Hounsfield Units, HU) were measured and subsequently tumor-to-parenchyma ratios calculated (Fig. 3b). Additionally, based on their attenuation, tumors were classified hypodense, isodense or hyperdense by comparison with the adjacent, non-involved pancreas parenchyma and also assigned to one of the two categories “homogeneously” versus “heterogeneously” attenuated.

### Statistical analysis

Statistical analysis was performed using SPSS Version 22 (IBM Corporation). We tested all parameters for the normality by using Kolmogorov–Smirnov test. A Mann–Whitney-U test was used to test the difference in textural features between the two groups (PDAC and PNEN). To address the multiple comparisons, a Benjamin Hochberg correction was applied. The adjusted p-values were considered significant at a level of 0.05.

On all parameters a z-transformation was applied followed by binary logistic regression analysis (forward LR stepwise method) using the most significant parameters to construct multi-indicator models for prediction of PDAC or PNEN. To test the significance of the logistic regression model a *χ*^2^ test was applied and the Cox & Snell-*R*^2^ was calculated. Receiver operating characteristic (ROC) analysis was performed to assess the predictive value by calculating the areas under the ROC curve (AUCs). The ROC curve was generated by computing sensitivity and specificity at each observed cut-off. The optimal cut-off values are derived from the point on the ROC curve with the minimum distance to the upper left corner (where sensitivity and specificity equal 1, respectively).

## Results

### Patient characteristics

53 patients (66.1 ± 8.6y, 24 female) with PDAC and 42 patients (65.5 ± 12.2y; 18 female) with PNEN were included. In PNEN, 21/42 patients had tumor grade II/III and 8/42 patients had tumor grade I. In 13 PNEN patients no information regarding tumor grading was available. In PDAC, 2/53 patients had tumor grade I, whereas 16/53 patients had tumor grade II or III. No information regarding tumor grading was available in 35 patients, most of them being sampled at other institutions. The clinical characteristics of all patients are summarized in Table 1.

**Table 1:** Patient characteristics

Characteristics	PDAC	PNEN
*N*	53	42
Age (years)		
Mean ± SD	66.1 ± 8.6	65.5 ± 12.2
Sex, *n* (%)		
Males	29 (54.7%)	24
Females	24 (45.3%)	18
pTNM-Stage		
T1	2	4
T2	4	6
T3	31	16
T4	5	2
Tx	11	14
N0	9	8
N1	27	19
Nx	17	15
M0	28	15
M1	9	23
Mx	16	4
Grading		
G1	2	8
G2	11	15
G3	5	6
Tumor localization		
Pancreas head	32	15
Pancreas body	15	11
Pancreas tail	6	16
Tumor size (Mean ± SD)	2.6 ± 0.9 cm	3.2 ± 1.8 cm

### Analysis of CT-textural features in PDAC and PNEN

**Fig. 4:**
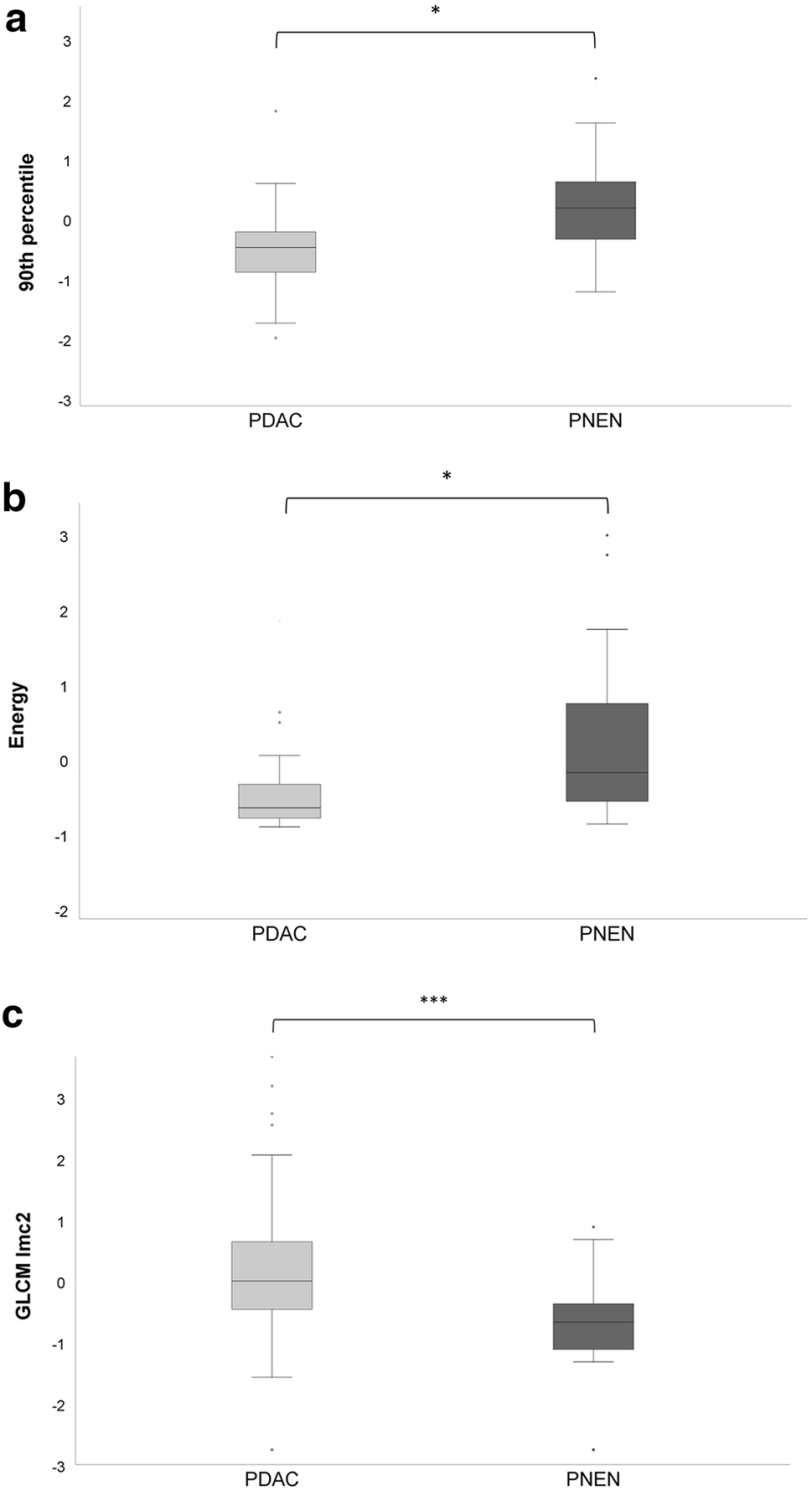
**a**–**c** Box plots showing the distribution of 1st order statistical features energy, 90th percentile and 2nd order gray-level co-occurrence matrix informational measure of correlation 2 in pancreatic adenocarcinoma (PDAC) and pancreatic neuroendocrine neoplasms (PNEN)

Of 92 textural features, 8 (8.7%) proved significantly different between PDAC and PNEN. The first-order features “median” and “maximum” were both significantly lower in PDAC (− 0.37 ± 1.07 [median]; − 0.21 ± 1.10 [maximum]) compared to PNEN (0.41 ± 0.77 [median], *p* = 0.0003; 0.27 ± 0.78 [maximum], *p* = 0.04). Complementary, the first-order feature “90th percentile” (Fig. 4a) proved significantly lower in PDAC (− 0.26 ± 1.10) than in PNEN (0.33 ± 0.74; *p* = 0.001), which was also observed for “10th percentile” (− 0.46 ± 0.99 [PDAC] vs. 0.45 ± 0.84 [PNEN]; *p* = 0.001). The other first-order features “energy” (*p* = 0.02), “total energy” (*p* = 0.0001), and “minimum” (*p* = 0.00002) were significantly higher in PNEN compared to PDAC with high standard deviations (Table 1; Fig. 4b). In contrast, the first-order “entropy” proved higher in PDAC (− 0.17 ± 1.26) compared to PNEN (− 0.33 ± 1.15), however, without reaching statistical significance (*p* > .05).

The 2nd order feature GLCM Imc2 was significantly higher in PDAC (− 0.03 ± 1.47) than in PNEN (− 0.89 ± 0.99; *p* = 0.0002) (Fig. 4c).

### Multivariate logistic regression analysis for classification of PDAC Versus PNEN

The logistic regression analysis including significantly different features between PDAC and PNEN resulted in a significant model (*χ*^2^(8) = 34.50; *p* < .001) with $r_{{{\text{Cox}}\;\& \;{\text{Snell}}}}^{2} = 0.30$, a Nagelkerke’s *r*^2^ = 0.41 and a Cohen’s effect size of $f = \sqrt {\frac{0.41}{1 - 0.41}} = 0.83$. 75.8% of patients had been classified correctly as PDAC or PNEN by the logistic model. 42/53 patients had been predicted correctly as PDAC (sensitivity 79.2%) and 12/42 patients had been predicted correctly as PNEN (sensitivity 71.4%).

**Fig. 5:**
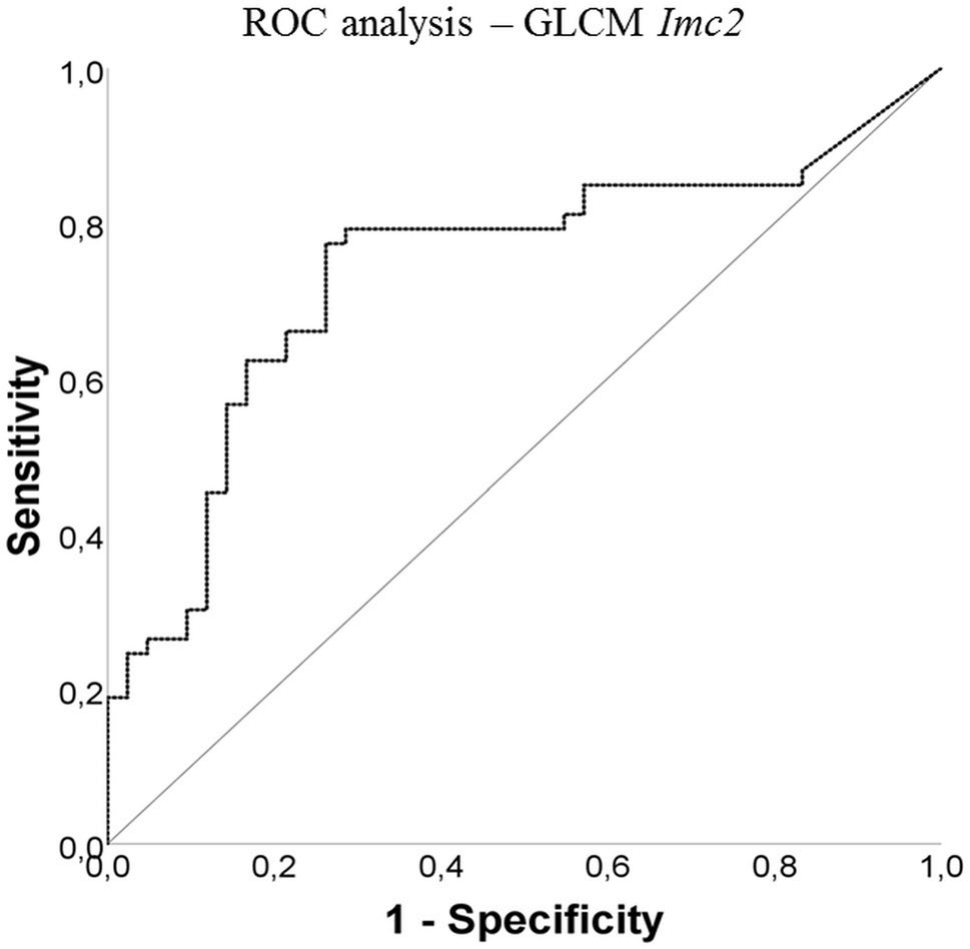
ROC analysis for gray-level co-occurrence matrix informational measure of correlation 2 for differentiation of PDAC from PNEN

In this multivariate logistic regression model, GLCM IMC2 proved to be the variable with the most impact on the odds ratio. The results containing all explanatory variables (full model) are shown in Table 2. For GLCM IMC2 the ROC analysis derived − 0.49 as a cut-off value to differentiate between PDAC and PNEN with a sensitivity of 0.79 and a specificity of 0.71 (Fig. 5).

**Table 2:** Results from multivariate logistic regression model containing all explanatory variables (full model)

Radiomic feature	Exp (*β*)	95% CI	*p* value
Median	0.42	0.002–86.78	0.75
Maximum	3.98	0.22–72.69	0.65
Minimum	1.35	0.20–9.08	0.76
10th percentile	5.66	0.16–197.44	0.34
90th percentile	0.28	0.001–52.34	0.63
Total energy	5.25	0.20–1407.36	0.56
Energy	0.27	0.001–74.59	0.27
GLCM Imc2	0.56	0.36–0.89	0.01

### Subgroup analysis of PDAC and PNEN depending on grading

**Fig. 6:**
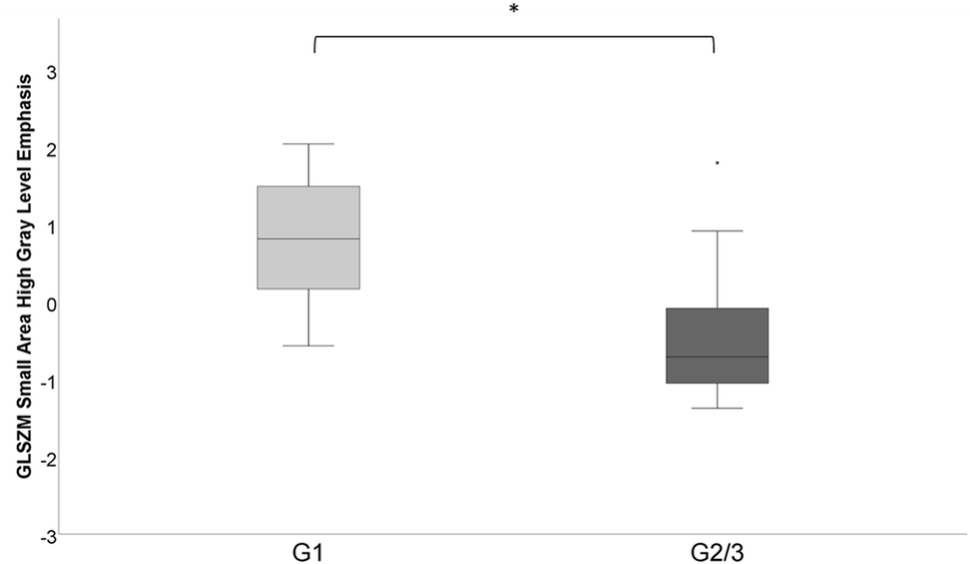
Box plots showing the distribution of gray-level size zone matrix [GLSZM] Small Area High Gray-Level Emphasis in pancreatic neuroendocrine neoplasms (PNEN) grade 1 versus grade 2/3

In PNEN, the higher order feature GLSZM Small Area High Gray-Level Emphasis proved significantly higher in patients with G1 (0.80 ± 0.90) compared to patients with G2/3 tumors (− 0.47 ± 0.86; *p* < .05) (Fig. 6). In PDAC, no significant differences in textural features between patients with G1 and G2/3 tumors were observed.

### Visual tumor classification and tumor-to-pancreas attenuation quantification

In the subgroup of patients with PDAC, the mean attenuation value was 73 ± 26 HU with a mean attenuation of pancreatic parenchyma of 85 ± 20 HU. The calculated tumor-to-pancreas ratio was 0.85.

In PNEN, we measured a mean attenuation value of 80.3 ± 19 HU, whereas the attenuation of the pancreas parenchyma was 88 ± 22 HU resulting in a tumor-to-parenchyma ratio of 0.90. Differences between the tumor-to-pancreas ratios measured in PDAC and PNEN did not reach statistical significance (*p* > 0.05).

In both PDAC and PNEN only 50% of the tumors exhibited homogeneous attenuation.

PDACs and PNENs were classified into hypo-, iso- or hyperdense in 50%/50%/0% and 45%/45%/10%, respectively (*p* > 0.05).

## Discussion

Our results show that CT-textural features quantified on portal-venous CT-image data are capable to differentiate between PDAC and PNEN. In fact, 8.7% of all analyzed statistical features proved discriminatory including 1st order and 2nd order variables. Interestingly, these features concentrate mainly on differences in the tumor attenuation (median, maximum, minimum, 10% and 90% percentile, energy, total energy) as well as on tumor structure (gray-level co-occurrence matrix) judged by tumor intensity histogram analysis (1st order) as well as on the second-order conditional probability density function.

These disparities quantified by means of textural (radiomics) features presumably reflect both distinctions in the composition of these tumors (cell size, density, amount of fibrotic stroma, etc.) and such related to the properties of the vascular network (microvessel density, irregularities in the shape and distribution of the glands, blood flow characteristics, vessel leakiness, intratumoral pressure) [20]. PDAC is known for its extensive desmoplastic reaction, whereas its vascular network is less well developed as in PNEN which has a highly vascularized tumor stroma [20, 21]. Previous reports using perfusion-CT demonstrated a 60% reduction in blood flow in PDAC compared to normal pancreas tissue [22]. D’assignies et al. reported a doubling of blood flow values in PNEN compared to the normal pancreas tissue [13]. Hence, differences in the amount of blood supply to PDAC versus PNEN are large and seem to play a major role in the distinction of these two entities even in the portal-venous phase which in this particular setting can be generally regarded as less specific. Notably, the entropy in PDAC was found higher compared to PNEN; however, this value did not reach statistical significance. Nevertheless, this result is in support of an increased complexity of the tissue texture in PDAC as evaluated by the gray-level co-occurrence matrix which proved significantly different to PNEN. This feature (Informational Measure of Correlation) assesses the correlation between the probability distributions of voxel values showing a more even texture in PNEN.

The idea of adding quantification to the qualitative visual imaging findings for more accurate characterization of pancreatic tumors is not new and different imaging techniques have been advocated for this task over the time [23–26]. Nevertheless, CT remains the working horse for primary imaging diagnosis and the idea of empowering the quality of this technique by adding image data quantification is attractive. In a recent report, Li et al. described differences in the magnitude of some of the 1st order radiomics features between PDAC and atypical neuroendocrine tumors [27]. In their analysis, mean, median, 5th, 10th, and 25th percentiles proved lower, whereas skewness proved higher in PDAC compared to atypical neuroendocrine tumors which is in line with most of our own results. Of note, these authors used a different post-processing tool, but a similar time delay for the portal-venous enhancement phase on which they applied textural analysis which suggests reproducibility of 1st order feature quantification. In a similar attempt, Toshikazu et al. using an MRI-approach found significantly higher entropy, skewness, and kurtosis in PDAC and higher means in neuroendocrine tumors by applying 1st order radiomics analysis on ADC-image data, which again are in support of their diagnostic value beyond the limits of individual imaging modalities [23].

A few other reports addressing the role of radiomics analysis in pancreatic tumors focused exclusively on the correlations between tumor grading and local tumor aggressiveness using different numbers of extracted textural features [15–18]. In particular, entropy was found to correlate well with the risk of early disease progression after surgical resection [28]. In a report by Choi et al., grade 2/3 pancreatic neuroendocrine tumors were more likely to show higher skewness, lower kurtosis, higher homogeneity, larger volume, and lower GLCM (gray-level co-occurrence matrix) moments [15]. D’Onofrio et al. found significant differences in the magnitude of entropy and kurtosis in pancreatic neuroendocrine tumors using radiomics analysis on CT-image data acquired in the portal-venous phase [16]. Indeed, the portal-venous enhancement phase has been recommended by many previous studies as comparable with the pancreatic phase for improved tissue contrast; however, this perception is questionable [29, 30]. In our cohort, the joint distribution of smaller size zones with higher gray-level values (proportion of the joint distribution of smaller size zones with higher gray-level values in the image) which is a measure of homogeneity was significantly higher (> double) in G1 PNEN versus G2/G3 PNEN. Focusing on the statistically significant textural feature of the 2nd order (co-occurrence matrix), results are suggesting a higher magnitude of higher-gray-level values and joint distribution in PNEN versus PDAC. Interestingly, we found no significant differences in terms of radiomics features among PDAC of different gradings.

Finally, our results showed great overlap between the two tumor entities in terms of visual assessment and even quantification of tumor-to-parenchyma ratios with no signal significant finding. Consequently, we believe that in such cases the additional use of CTTA could improve diagnostic quality delivering complementary information without the need for subsequent additional imaging which might improve patient management (e.g., staging procedures).

Our study has some limitations. First, our image data were collected on different multi-slice scanner, but using a similar examination and contrast agent injection protocol. Nevertheless, some variations in image quality are inherently expected. This aspect should stress also the applicability of textural analysis on different image data sets with comparable results in our cohort. Second, morphologic imaging features (e.g., form, size, contours) were not evaluated in this cohort as we considered that there is already enough evidence on this topic in the current specialty literature.

In conclusion, our data indicate that CT-texture analysis is a feasible tool for differentiation of PNEN from PDAC and also of G1 from G2/3 PNEN in the portal-venous phase. Most textural features reflect lower tissue attenuation and uniformity in PDAC as compared to PNEN. Notably, CTTA seems to outmatch the results of both visual assessment and tumor attenuation quantification.

## Electronic supplementary material

Below is the link to the electronic supplementary material.

Supplementary material 1 (DOCX 47 kb)
