# Correction to: Complementary role of computed tomography texture analysis for differentiation of pancreatic ductal adenocarcinoma from pancreatic neuroendocrine tumors in the portal‑venous enhancement phase

**DOI:** 10.1007/s00261-021-03084-x

**Published:** 2021-05-07

**Authors:** Christian Philipp Reinert, Karolin Baumgartner, Tobias Hepp, Michael Bitzer, Marius Horger

**Affiliations:** 1 Department of Diagnostic and Interventional Radiology, University Hospital Tübingen, Hoppe-Seyler-Str. 3, 72076 Tübingen, Germany; 2 Department of Internal Medicine I, Hepatology, Gastroenterology, Infectiology, University Hospital Tübingen, Otfried-Müller-Str. 10, 72076 Tübingen, Germany

## Correction to: Abdominal Radiology (2020) 45:750–758 10.1007/s00261-020-02406-9

The article “Complementary role of computed tomography texture analysis for differentiation of pancreatic ductal adenocarcinoma from pancreatic neuroendocrine tumors in the portal‑venous enhancement phase”, written by Christian Philipp Reinert, Karolin Baumgartner, Tobias Hepp, Michael Bitzer and Marius Horger, was originally published electronically on the publisher's internet portal on 17 January 2020 without open access. With the author(s)' decision to opt for Open Choice the copyright of the article changed on 1 April 2021 to © The Author(s) 2020 and the article is forthwith distributed under a Creative Commons Attribution 4.0 Inter-national License, which permits use, sharing, adaptation, distribution and reproduction in any medium or format, as long as you give appropriate credit to the original author(s) and the source, provide a link to the Creative Commons licence, and indicate if changes were made. The images or other third party material in this article are included in the article's Creative Commons licence, unless indicated otherwise in a credit line to the material. If material is not included in the article's Creative Commons licence and your intended use is not permitted by statutory regulation or exceeds the permitted use, you will need to obtain per-mission directly from the copyright holder. To view a copy of this licence, visit https://creativecommons.org/licenses/by/4.0.

The original article has been corrected.

