# Supplementary material for: Complementary role of computed tomography texture analysis for differentiation of pancreatic ductal adenocarcinoma from pancreatic neuroendocrine tumors in the portal-venous enhancement phase
Source: Abdom Radiol (NY). 2020 Jan 17;45(3):750–8. doi: 10.1007/s00261-020-02406-9 (PMC8081676; doi:10.1007/s00261-020-02406-9)
Supplement: Supplementary file 1 — Supplementary material 1 (DOCX 47 kb) [file 261_2020_2406_MOESM1_ESM.docx]

**Table 3.** Complete listing of all measured features categorised according to their classes

| **Classes** | **Feature abbreviation** | **Definition** |
| --- | --- | --- |
| First-order statistics  Describe the distribution of voxel intensities within the image region defined by the mask through commonly used and basic metrics. | original_firstorder_10Percentile | 10th percentile |
|  | original_firstorder_90Percentile | 90th percentile |
|  | original_firstorder_Energy | Energy is a measure of the magnitude of voxel values in an image. A larger value implies a greater sum of the squares of these values. |
|  | original_firstorder_Entropy | Entropy specifies the uncertainty/randomness in the image values. It measures the average amount of information required to encode the image values. |
|  | original_firstorder_InterquartileRange | 25th and 75th percentile of the image array |
|  | original_firstorder_Kurtosis | Kurtosis is a measure of the ‘peakedness’ of the distribution of values in the image ROI. A higher kurtosis implies that the mass of the distribution is concentrated towards the tail(s) rather than towards the mean. A lower kurtosis implies the reverse: that the mass of the distribution is concentrated towards a spike near the Mean value. |
|  | original_firstorder_Maximum | maximum |
|  | original_firstorder_Mean | mean |
|  | original_firstorder_MeanAbsoluteDeviation | Mean Absolute Deviation is the mean distance of all intensity values from the Mean Value of the image array. |
|  | original_firstorder_Median | median |
|  | original_firstorder_Minimum | minimum |
|  | original_firstorder_Range | The range of gray values in the ROI. |
|  | original_firstorder_RobustMeanAbsoluteDeviation | Robust Mean Absolute Deviation is the mean distance of all intensity values from the Mean Value calculated on the subset of image array with gray levels in between, or equal to the 10th and 90th percentile. |
|  | original_firstorder_RootMeanSquared | Root Mean Squared (RMS) is the square-root of the mean of all the squared intensity values. It is another measure of the magnitude of the image values. |
|  | original_firstorder_Skewness | Skewness measures the asymmetry of the distribution of values about the Mean value. Depending on where the tail is elongated and the mass of the distribution is concentrated, this value can be positive or negative. |
|  | original_firstorder_TotalEnergy | Total Energy is the value of Energy feature scaled by the volume of the voxel in cubic mm. |
|  | original_firstorder_Uniformity | Uniformity is a measure of the sum of the squares of each intensity value. This is a measure of the homogeneity of the image array, where a greater uniformity implies a greater homogeneity or a smaller range of discrete intensity values. |
|  | original_firstorder_Variance | Variance is the the mean of the squared distances of each intensity value from the Mean value. This is a measure of the spread of the distribution about the mean. |
| Gray Level Co-occurrence Matrix  Describes the second order joint probability function for the given region of co-occurring pixel intensities | original_glcm_Autocorrelation | Autocorrelation is a measure of the magnitude of the fineness and coarseness of texture. |
|  | original_glcm_ClusterProminence | Cluster Prominence is a measure of the skewness and asymmetry of the GLCM. A higher value implies more asymmetry about the mean while a lower value indicates a peak near the mean value and less variation about the mean. |
|  | original_glcm_ClusterShade | Cluster Shade is a measure of the skewness and uniformity of the GLCM. A higher cluster shade implies greater asymmetry about the mean. |
|  | original_glcm_ClusterTendency | Cluster Tendency is a measure of groupings of voxels with similar gray-level values. |
|  | original_glcm_Contrast | Contrast is a measure of the local intensity variation, favoring values away from the diagonal. A larger value correlates with a greater disparity in intensity values among neighboring voxels. |
|  | original_glcm_Correlation | Correlation is a value between 0 (uncorrelated) and 1 (perfectly correlated) showing the linear dependency of gray level values to their respective voxels in the GLCM. |
|  | original_glcm_DifferenceAverage | Difference Average measures the relationship between occurrences of pairs with similar intensity values and occurrences of pairs with differing intensity values. |
|  | original_glcm_DifferenceEntropy | Difference Entropy is a measure of the randomness/variability in neighborhood intensity value differences. |
|  | original_glcm_DifferenceVariance | Difference Variance is a measure of heterogeneity that places higher weights on differing intensity level pairs that deviate more from the mean. |
|  | original_glcm_Id | Inverse Difference (ID) (a.k.a. Homogeneity 1) is another measure of the local homogeneity of an image. With more uniform gray levels, the denominator will remain low, resulting in a higher overall value. |
|  | original_glcm_Idm | Inverse Difference Moment (IDM) (a.k.a Homogeneity 2) is a measure of the local homogeneity of an image. IDM weights are the inverse of the Contrast weights (decreasing exponentially from the diagonal i=j in the GLCM). |
|  | original_glcm_Idmn | Inverse Difference Moment Normalized (IDMN) IDMN (inverse difference moment normalized) is a measure of the local homogeneity of an image. IDMN weights are the inverse of the Contrast weights (decreasing exponentially from the diagonal i=j in the GLCM). Unlike Homogeneity2, IDMN normalizes the square of the difference between neighboring intensity values by dividing over the square of the total number of discrete intensity values. |
|  | original_glcm_Idn | Inverse Difference Normalized (IDN) is another measure of the local homogeneity of an image. Unlike Inverse Difference, IDN normalizes the difference between the neighboring intensity values by dividing over the total number of discrete intensity values. |
|  | original_glcm_Imc1 | Informational Measure of Correlation (IMC) 1 assesses the correlation between the probability distributions of i and j (quantifying the complexity of the texture), using mutual information I(x, y). |
|  | original_glcm_Imc2 | Informational Measure of Correlation (IMC) 2 also assesses the correlation between the probability distributions of i and j (quantifying the complexity of the texture). |
|  | original_glcm_InverseVariance | Inverse Variance of GLCM. |
|  | original_glcm_JointAverage | Joint average returns the mean gray level intensity of the i distribution. |
|  | original_glcm_JointEnergy | Energy is a measure of homogeneous patterns in the image. A greater Energy implies that there are more instances of intensity value pairs in the image that neighbor each other at higher frequencies. |
|  | original_glcm_JointEntropy | Joint entropy is a measure of the randomness/variability in neighborhood intensity values. |
|  | original_glcm_MaximumProbability | Maximum Probability is occurrences of the most predominant pair of neighboring intensity values. |
|  | original_glcm_SumAverage | Sum Average measures the relationship between occurrences of pairs with lower intensity values and occurrences of pairs with higher intensity values. |
|  | original_glcm_SumEntropy | Sum Entropy is a sum of neighborhood intensity value differences. |
|  | original_glcm_SumSquares | Sum of Squares or Variance is a measure in the distribution of neigboring intensity level pairs about the mean intensity level in the GLCM. |
| Gray level Dependence Matrix  Quantifies gray level dependencies in an image. A gray level dependency is defined as a the number of connected voxels within distance δ that are dependent on the center voxel. | original_gldm_DependenceEntropy | Dependence Entropy (DE) measures the entropy in dependence size in the image. |
|  | original_gldm_DependenceNonUniformity | Dependence Non-Uniformity (DN) measures the similarity of dependence throughout the image, with a lower value indicating more homogeneity among dependencies in the image. |
|  | original_gldm_DependenceNonUniformityNormalized | Dependence Non-Uniformity Normalized (DNN) measures the similarity of dependence throughout the image, with a lower value indicating more homogeneity among dependencies in the image. |
|  | original_gldm_DependenceVariance | Dependence Variance (DV) measures the variance in dependence size in the image. |
|  | original_gldm_GrayLevelNonUniformity | Gray Level Non-Uniformity (GLN) measures the similarity of gray-level intensity values in the image, where a lower GLN value correlates with a greater similarity in intensity values. |
|  | original_gldm_GrayLevelVariance | Gray Level Variance (GLV) measures the variance in grey level in the image. |
|  | original_gldm_HighGrayLevelEmphasis | High Gray Level Emphasis (HGLE) measures the distribution of the higher gray-level values, with a higher value indicating a greater concentration of high gray-level values in the image. |
|  | original_gldm_LargeDependenceEmphasis | Large Dependence Emphasis (LDE) is a measure of the distribution of large dependencies, with a greater value indicative of larger dependence and more homogeneous textures. |
|  | original_gldm_LargeDependenceHighGrayLevelEmphasis | Large Dependence High Gray Level Emphasis (LDHGLE) measures the joint distribution of large dependence with higher gray-level values. |
|  | original_gldm_LargeDependenceLowGrayLevelEmphasis | Large Dependence Low Gray Level Emphasis (LDLGLE) measures the joint distribution of large dependence with lower gray-level values. |
|  | original_gldm_LowGrayLevelEmphasis | Low Gray Level Emphasis (LGLE) measures the distribution of low gray-level values, with a higher value indicating a greater concentration of low gray-level values in the image. |
|  | original_gldm_SmallDependenceEmphasis | Small Dependence Emphasis (SDE) is a measure of the distribution of small dependencies, with a greater value indicative of smaller dependence and less homogeneous textures. |
|  | original_gldm_SmallDependenceHighGrayLevelEmphasis | Small Dependence High Gray Level Emphasis (SDHGLE) measures the joint distribution of small dependence with higher gray-level values. |
|  | original_gldm_SmallDependenceLowGrayLevelEmphasis | |
| Gray Level Run Length Matrix  Quantifies gray level runs, which are defined as the length in number of pixels, of consecutive pixels that have the same gray level value. | original_glrlm_GrayLevelNonUniformity | Gray Level Non-Uniformity (GLN) measures the similarity of gray-level intensity values in the image, where a lower GLN value correlates with a greater similarity in intensity values. |
|  | original_glrlm_GrayLevelNonUniformityNormalized | Gray Level Non-Uniformity Normalized (GLNN) measures the similarity of gray-level intensity values in the image, where a lower GLNN value correlates with a greater similarity in intensity values. |
|  | original_glrlm_GrayLevelVariance | Gray Level Variance (GLV) measures the variance in gray level intensity for the runs. |
|  | original_glrlm_HighGrayLevelRunEmphasis | High Gray Level Run Emphasis (HGLRE) measures the distribution of the higher gray-level values, with a higher value indicating a greater concentration of high gray-level values in the image. |
|  | original_glrlm_LongRunEmphasis | Long Run Emphasis (LRE) is a measure of the distribution of long run lengths, with a greater value indicative of longer run lengths and more coarse structural textures. |
|  | original_glrlm_LongRunHighGrayLevelEmphasis | Long Run High Gray Level Emphasis (LRHGLE) measures the joint distribution of long run lengths with higher gray-level values. |
|  | original_glrlm_LongRunLowGrayLevelEmphasis | Long Run Low Gray Level Emphasis (LRLGLE) measures the joint distribution of long run lengths with lower gray-level values. |
|  | original_glrlm_LowGrayLevelRunEmphasis | Low Gray Level Run Emphasis (LGLRE) measures the distribution of low gray-level values, with a higher value indicating a greater concentration of low gray-level values in the image. |
|  | original_glrlm_RunEntropy | Run Entropy (RE) measures the uncertainty/randomness in the distribution of run lengths and gray levels. A higher value indicates more heterogeneity in the texture patterns. |
|  | original_glrlm_RunLengthNonUniformity | Run Length Non-Uniformity (RLN) measures the similarity of run lengths throughout the image, with a lower value indicating more homogeneity among run lengths in the image. |
|  | original_glrlm_RunLengthNonUniformityNormalized | Run Length Non-Uniformity Normalized (RLNN) measures the similarity of run lengths throughout the image, with a lower value indicating more homogeneity among run lengths in the image. |
|  | original_glrlm_RunPercentage | Run Percentage (RP) measures the coarseness of the texture by taking the ratio of number of runs and number of voxels in the ROI. |
|  | original_glrlm_RunVariance | Run Variance (RV) is a measure of the variance in runs for the run lengths. |
|  | original_glrlm_ShortRunEmphasis | Short Run Emphasis (SRE) is a measure of the distribution of short run lengths, with a greater value indicative of shorter run lengths and more fine textural textures. |
|  | original_glrlm_ShortRunHighGrayLevelEmphasis | Short Run High Gray Level Emphasis (SRHGLE) measures the joint distribution of shorter run lengths with higher gray-level values. |
|  | original_glrlm_ShortRunLowGrayLevelEmphasis | Short Run Low Gray Level Emphasis (SRLGLE) measures the joint distribution of shorter run lengths with lower gray-level values. |
| Gray Level Size Zone (GLSZM)  quantifies gray level zones in an image. A gray level zone is defined as a the number of connected voxels that share the same gray level intensity. A voxel is considered connected if the distance is 1 according to the infinity norm (26-connected region in a 3D, 8-connected region in 2D). | original_glszm_GrayLevelNonUniformity | Gray Level Non-Uniformity (GLN) measures the variability of gray-level intensity values in the image, with a lower value indicating more homogeneity in intensity values. |
|  | original_glszm_GrayLevelNonUniformityNormalized | Gray Level Non-Uniformity Normalized (GLNN) measures the variability of gray-level intensity values in the image, with a lower value indicating a greater similarity in intensity values. |
|  | original_glszm_GrayLevelVariance | Gray Level Variance (GLV) easures the variance in gray level intensities for the zones. |
|  | original_glszm_HighGrayLevelZoneEmphasis | High Gray Level Zone Emphasis (HGLZE) measures the distribution of the higher gray-level values, with a higher value indicating a greater proportion of higher gray-level values and size zones in the image. |
|  | original_glszm_LargeAreaEmphasis | Large Area Emphasis (LAE) is a measure of the distribution of large area size zones, with a greater value indicative of more larger size zones and more coarse textures. |
|  | original_glszm_LargeAreaHighGrayLevelEmphasis | Large Area High Gray Level Emphasis (LAHGLE) measures the proportion in the image of the joint distribution of larger size zones with higher gray-level values. |
|  | original_glszm_LargeAreaLowGrayLevelEmphasis | Large Area Low Gray Level Emphasis (LALGLE) measures the proportion in the image of the joint distribution of larger size zones with lower gray-level values. |
|  | original_glszm_LowGrayLevelZoneEmphasis | Low Gray Level Zone Emphasis (LGLZE) measures the distribution of lower gray-level size zones, with a higher value indicating a greater proportion of lower gray-level values and size zones in the image. |
|  | original_glszm_SizeZoneNonUniformity | Size-Zone Non-Uniformity (SZN) measures the variability of size zone volumes in the image, with a lower value indicating more homogeneity in size zone volumes. |
|  | original_glszm_SizeZoneNonUniformityNormalized | Size-Zone Non-Uniformity Normalized (SZNN) measures the variability of size zone volumes throughout the image, with a lower value indicating more homogeneity among zone size volumes in the image. |
|  | original_glszm_SmallAreaEmphasis | Small Area Emphasis (SAE) is a measure of the distribution of small size zones, with a greater value indicative of more smaller size zones and more fine textures. |
|  | original_glszm_SmallAreaHighGrayLevelEmphasis | Small Area High Gray Level Emphasis (SAHGLE) measures the proportion in the image of the joint distribution of smaller size zones with higher gray-level values. |
|  | original_glszm_SmallAreaLowGrayLevelEmphasis | Small Area Low Gray Level Emphasis (SALGLE) measures the proportion in the image of the joint distribution of smaller size zones with lower gray-level values. |
|  | original_glszm_ZoneEntropy | Zone Entropy (ZE) measures the uncertainty/randomness in the distribution of zone sizes and gray levels. A higher value indicates more heterogeneneity in the texture patterns. |
|  | original_glszm_ZonePercentage | Zone Percentage (ZP) measures the coarseness of the texture by taking the ratio of number of zones and number of voxels in the ROI. |
|  | original_glszm_ZoneVariance | Zone Variance (ZV) measures the variance in zone size volumes for the zones. |
| Neighbouring Gray Tone Difference Matrix  Quantifies the difference between a gray value and the average gray value of its neighbours within distance δ. | original_ngtdm_Busyness | Busyness is a measure of the change from a pixel to its neighbour. A high value for busyness indicates a ‘busy’ image, with rapid changes of intensity between pixels and its neighbourhood. |
|  | original_ngtdm_Coarseness | Coarseness is a measure of average difference between the center voxel and its neighbourhood and is an indication of the spatial rate of change. A higher value indicates a lower spatial change rate and a locally more uniform texture. |
|  | original_ngtdm_Complexity | Complexity: An image is considered complex when there are many primitive components in the image, i.e. the image is non-uniform and there are many rapid changes in gray level intensity. |
|  | original_ngtdm_Contrast | Contrast is a measure of the spatial intensity change, but is also dependent on the overall gray level dynamic range. Contrast is high when both the dynamic range and the spatial change rate are high, i.e. an image with a large range of gray levels, with large changes between voxels and their neighbourhood. |
|  | original_ngtdm_Strength | Strenght is a measure of the primitives in an image. Its value is high when the primitives are easily defined and visible, i.e. an image with slow change in intensity but more large coarse differences in gray level intensities. |
